# Neuropsychiatric Disorder Associated with Group G *Streptococcus* Infection

**DOI:** 10.1155/2018/6047318

**Published:** 2018-09-23

**Authors:** Rie Okumura, Sawako Yamazaki, Tsukasa Ohashi, Shinichi Magara, Jun Tohyama, Hiroshi Sakuma, Masaharu Hayashi, Akihiko Saitoh

**Affiliations:** ^1^Department of Pediatrics, Niigata City General Hospital, Niigata, Japan; ^2^Department of Pediatrics, Niigata Prefecture Hamagumi Medical Rehabilitation Center for Disabled Children, Niigata, Japan; ^3^Department of Pediatrics, Niigata Minami Hospital, Niigata, Japan; ^4^Department of Child Neurology, Nishi-Niigata Chuo National Hospital, Niigata, Japan; ^5^Department of Brain Development and Neural Regeneration, Tokyo Metropolitan Institute of Medical Science, Tokyo, Japan; ^6^School of Nursing, College of Nursing and Nutrition, Shukutoku University, Chiba, Japan; ^7^Department of Pediatrics, Niigata University, Niigata, Japan

## Abstract

Immune-mediated central nervous system manifestations of group A *β*-hemolytic *Streptococcus* (GABHS) infection include Sydenham's chorea, pediatric autoimmune neuropsychiatric disorders associated with streptococcal infection (PANDAS)—which includes tic and obsessive compulsive disorders—and a variety of neurobehavioral disorders. We report a case of *Streptococcus dysgalactiae* subspecies *equisimilis* (group G *Streptococcus*) (GGS) infection associated with involuntary movements, complex tics, and emotional lability in an 11-year-old Japanese girl. Serum IgM and IgG antibodies to lysoganglioside were positive, and she responded rapidly to intravenous immunoglobulin treatment. Neuropsychiatric disorder associated with GGS infection was ultimately diagnosed. The present findings suggest that neuropsychiatric disorders can result from GGS infection and that the pathogenic mechanism is similar to that of GABHS infection. Future large-scale studies should examine the relation between GGS infection and onset of neuropsychiatric disorder.

## 1. Introduction

Infection with group A *β*-hemolytic *Streptococcus* (GABHS) causes autoimmune disorders of the basal ganglia in children, such as Sydenham's chorea (SC) and pediatric autoimmune neuropsychiatric disorders associated with streptococcal infection (PANDAS) [1]. Non-GABHS infection, particularly with Lancefield groups C and G, has been identified as a potential cause of acute pharyngitis in children and adults [2]. Accumulating evidence indicates that group G *Streptococcus* (GGS) and GABHS cause infection by similar mechanisms [3]. Because GGS is evolutionarily closely related to GABHS, GGS may be capable of causing rheumatic fever [4]; however, few reports have described neuropsychiatric disorders, including Sydenham's chorea, caused by GGS. We report the first case of neuropsychiatric disorder associated with GGS, in an 11-year-old girl.

## 2. Case Report

An 11-year-old girl presenting with involuntary movements in the face and extremities, clumsiness, and slurred speech was admitted to our hospital. She had no family history of neuropsychiatric disorders. Early psychomotor development was normal, although mild mental retardation was suspected at school. At age 10 years, she developed transient vocal tics. About 3 months before admission, she had episodes of choreiform movements. There were no events preceding these symptoms. The symptoms gradually worsened to include dropping eating utensils, and her body weight decreased by 4 kg in 3 months because of difficulty in eating. About a week before admission, she could not walk without assistance and did not attend school, because of gait difficulties. These symptoms were not observed during sleep. There was no indication of recent infection, and she had no history of fever during the 3 months before admission.

On examination, she exhibited notable choreoathetoid movements of the face and extremities. She was unable to walk without assistance. Muscle cramping in the cheeks and palpebrae-like tics were observed. She was alert and cooperative with the examiners, and her orientation was maintained. However, she exhibited emotional lability, sudden loud vocalizations, and resistance to restraint by caregivers. She showed severe irritability, and rage attacks were circumscribed. Muscle tonus and deep tendon reflexes were normal.

**Figure 1:**
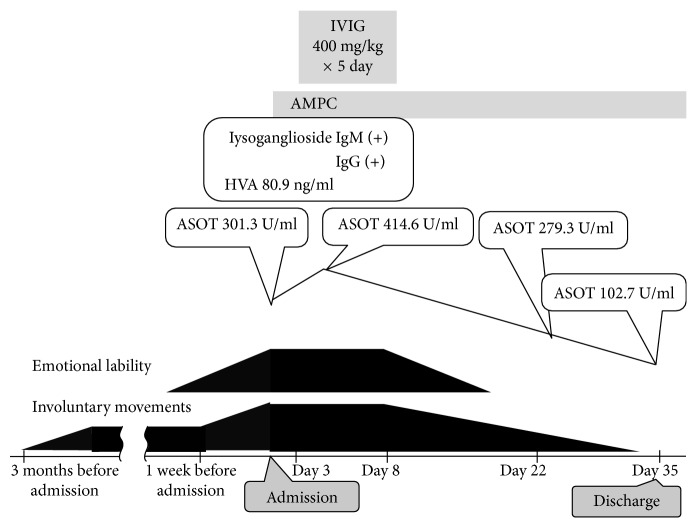
The patient's clinical course. IVIG, intravenous immunoglobulin; AMPC, amoxicillin; HVA, homovanillic acid; ASOT, antistreptolysin O titer.

Blood testing showed no abnormalities. Antistreptolysin O titer (ASOT) (301.3 IU/ml; normal, 0–330 IU/ml) and thyroid studies on admission were normal, but ASOT was mildly elevated (414.6 IU/ml) at 8 days after admission (Figure 1). GGS was isolated in a throat culture. Tests for rheumatoid factor, antinuclear antibody, and anticardiolipin antibody yielded negative results. Examination of cerebrospinal fluid (CSF) showed no pleocytosis or increase in protein level. Homovanillic acid (HVA) level in CSF was mildly elevated, at 80.9 ng/ml (normal, <50 ng/ml). Brain magnetic resonance imaging and electroencephalography findings were unremarkable. Serum IgM and IgG antibodies to lysoganglioside were positive. In SC and PANDAS, antilysoganglioside antibodies react with the neuronal cell surface because of a cross-reactive immune response with streptococcal antigen *N*-acetyl-beta-D-glucosamine (GlcNAc) [1, 5]. She was treated with oral ampicillin and intravenous immunoglobulin (IVIG) (400 mg/kg/day) for 5 days, and her symptoms eventually resolved. Her overall intelligence quotient on the Wechsler Intelligence Scale for Children, Third Edition was 50, which indicates mild intellectual disability.

## 3. Discussion

Immune-mediated central nervous system manifestations of GABHS infection include SC, PANDAS, and a variety of neurobehavioral disorders. Our patient's involuntary movements suddenly worsened. GGS was identified in a throat culture, and serial change in ASOT level suggests she was infected with GGS before exacerbation of her involuntary movements, which were probably caused by a postinfectious immune-mediated process. Our patient had an elevated HVA level in CSF. The HVA level is elevated in neuropsychiatric disorders, such as PANDAS, because dysfunction in dopaminergic neurotransmission in the mesolimbic pathway is involved in neuropsychiatric disorders [1]. It was unclear if GGS was colonized. However, because immunological testing showed antibodies to lysoganglioside, GGS infection likely caused the exacerbation of her involuntary movements, which resembled those of GABHS infection.

GGS is closely evolutionarily related to GABHS. They share many virulence factors, including hemolysis, extracellular enzymes, and M proteins. GGS produces M protein, which has structural, immunochemical, and biological features like those of the M protein of GABHS [2, 3]. GGS was reported to cause infections similar to GABHS, such as pharyngitis, skin and soft tissue infection, sepsis, toxic shock, reactive arthritis, and postglomerulonephritis [3, 6]. Although evidence is limited [7], the extensive homology between GGS and GABHS suggests that GGS may induce neuropsychiatric disorders that resemble the complications of GABHS.

Neuropsychiatric disorders like SC are believed to result from an immune-mediated response to GABHS infection [8]. Autoantibodies specific for lysoganglioside were detected in sera of patients with neuropsychiatric disorders like SC and PANDAS [5] and in our patient. SC may result from a cross-reactive, antistreptococcal antibody response directed against antigens of the basal ganglia, the area of the brain responsible for motor function, which suggests that neurological dysfunction in SC is immunologically mediated. Antibodies from SC and PANDAS recognized brain-derived lysoganglioside GM1 and GlcNAc [5]. We hypothesize that the neuropsychiatric symptoms of our patient were related to specific autoantibodies. In addition, antibody against dopmine-2 receptor is related to basal ganglia encephalitis associated with GABHS infection [9], although this antibody was not evaluated in our patient. She rapidly responded to IVIG treatment, which is effective in alleviating involuntary movements in patients with PANDAS and SC [10].

In conclusion, the present findings suggest that GGS infection can cause neuropsychiatric disorders; however, this hypothesis requires confirmation in future clinical studies.
